# Precision-cut rat placental slices as a model to study sex-dependent inflammatory response to LPS and Poly I:C

**DOI:** 10.3389/fimmu.2022.1083248

**Published:** 2022-12-20

**Authors:** Kasin Yadunandam Anandam, Cilia Abad, Tetiana Synova, Mireia Vinas-Noguera, Bahareh Bolboli, Ivan Vokral, Rona Karahoda, Frantisek Staud

**Affiliations:** Department of Pharmacology and Toxicology, Faculty of Pharmacy in Hradec Kralove, Charles University, Hradec Kralove, Czechia

**Keywords:** precision-cut slices, rat placenta, immune response, cytokines, LPS, Poly I:C

## Abstract

**Introduction:**

Maternal inflammation in pregnancy represents a major hallmark of several pregnancy complications and a significant risk factor for neurodevelopmental and neuropsychiatric disorders in the offspring. As the interface between the mother and the fetus, the placenta plays a crucial role in fetal development and programming. Moreover, studies have suggested that the placenta responds to an inflammatory environment in a sex-biased fashion. However, placenta-mediated immunoregulatory mechanisms are still poorly understood.

**Methods:**

Therefore, we have developed a model of *ex vivo* precision-cut placental slices from the rat term placenta to study acute inflammatory response. Rat placental slices with a precise thickness of 200 µm were generated separately from male and female placentas. Inflammation was stimulated by exposing the slices to various concentrations of LPS or Poly I:C for 4 and 18 hours.

**Results:**

Treatment of placental slices with LPS significantly induced the expression and release of proinflammatory cytokines TNF-α, IL-6, and IL-1β. In contrast, Poly I:C treatment resulted in a less-pronounced inflammatory response. Interestingly, the female placenta showed higher sensitivity to LPS than male placenta. Anti-inflammatory agents, curcumin, 1α,25- dihydroxyvitamin D3, and progesterone attenuated the LPS-induced proinflammatory cytokine response at both mRNA and protein levels.

**Discussion:**

We conclude that rat placental slices represent a novel alternative model to study the role of sexual dimorphism in the acute inflammatory response and immune activation in pregnancy.

## Introduction

The placenta is a versatile transient organ; in addition to its multiple roles in the exchange of gas, nutrients, and waste products between the maternal and fetal circulations, the placenta produces metabolites and hormones essential for fetal growth and development. Furthermore, the placenta acts as an immunological barrier between the maternal immune system and the fetus (1–3). The placenta undergoes dynamic physiological and structural changes throughout gestation to ensure proper fetal development and programming. Perturbations in placental formation and function by external insults, such as inflammation, pharmacotherapy, malnutrition, and environmental signals, can cause pregnancy complications and affect fetal programming (2).

Maternal inflammation has been associated with several pregnancy pathologies, including preterm birth, preeclampsia, and intrauterine growth restriction. It represents an important risk factor for neurodevelopmental and neuropsychiatric disorders in the offspring (4, 5). These pregnancy-related disorders are characterized by elevated levels of proinflammatory cytokines in maternal serum and placenta (4, 6). Maternal inflammation activates the production of proinflammatory cytokines, such as TNF-α, IL-6, IL-1β, and IFN-γ, that contribute to the risk of neuropsychiatric disorders through developmental failure. Although cytokines play an important role in normal fetal development, their aberrant levels may cause developmental defects in multiple organs, including the placenta, brain, heart, and lung (6). This is principally due to the direct transplacental transfer of maternal cytokines across the placenta (7, 8). In this regard, proinflammatory cytokines have emerged as a trigger for modulating several pathways in the placenta, including the development of dopamine- and serotonin-dependent neurogenic pathways and tryptophan metabolism (9–11). Interestingly, placental response to maternal inflammation is reportedly sex-dependent, although controversy exists in the published literature (12–15). Thus, further research is required to understand the molecular mechanisms behind the placental immune response to inflammation and the role of fetal sex.

**Figure 1:**
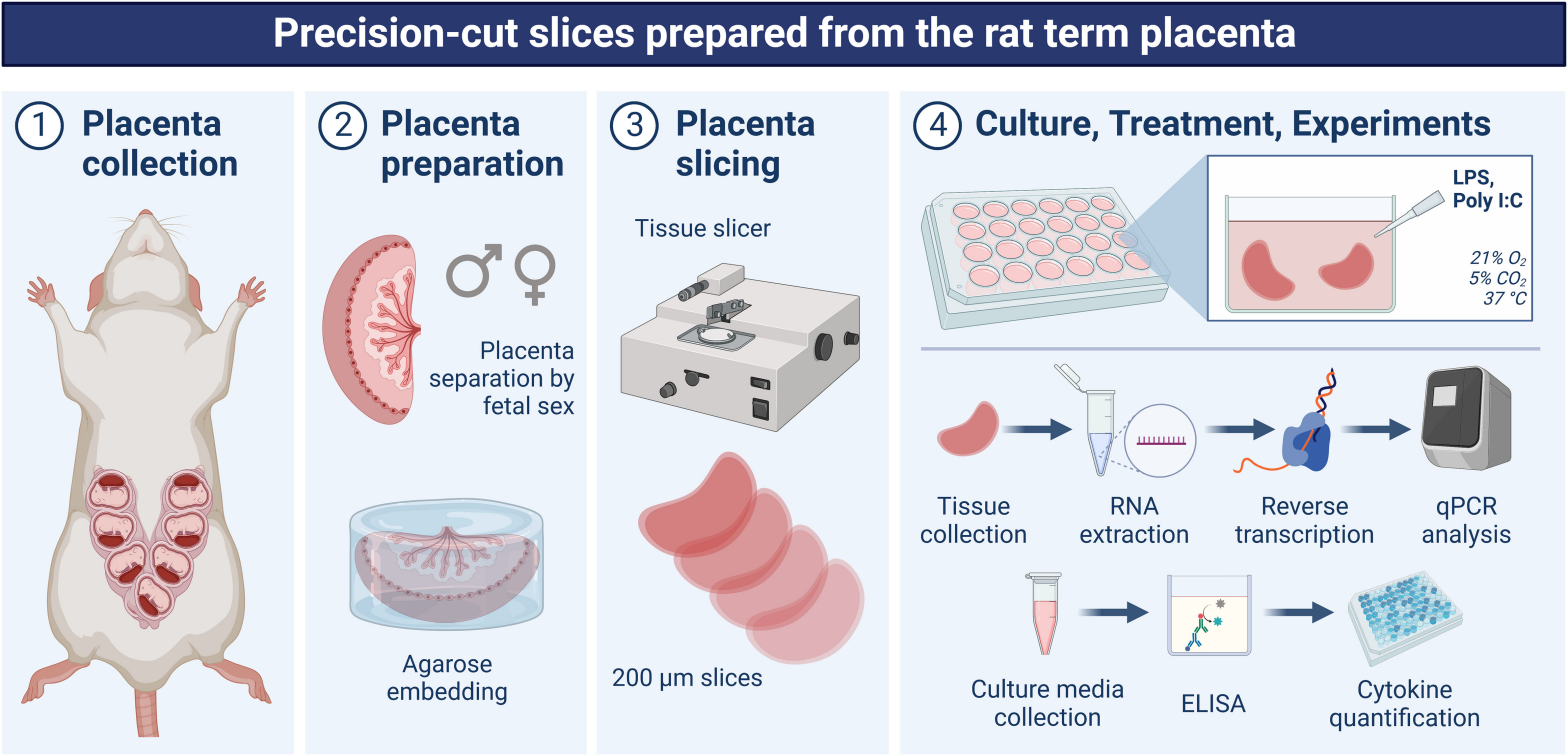
Schematic depiction of the experimental design used in this study. On gestation day 20, rats were anesthetized, and the uterine horns were exposed using a midline abdominal incision. Gestational sacs were separated, and placental samples were divided based on the fetal sex. Whole placentas were embedded in agarose, and placental slices with a thickness of 200 µm were generated using a sterilizable microtome. Slices were cultured in 24-well plates for 36 hours (stabilization period) and then exposed to various concentrations of LPS (0.1, 1, 5 µg/ml) or Poly I:C (1, 10, 50 µg/ml) for 4 and 18 hours. After the treatment, the tissue and the culture media were collected for qPCR and ELISA measurements, respectively. Created in BioRender.com.

Recent research has shown several benefits of precision-cut slices to investigate inflammatory response in various tissues, including the lungs, liver, and intestine (16–20). However, to date, no attempt has been made to employ this technique in exploring acute inflammatory response in the placenta. The placental slices contain the diverse cell types that characterize the placenta, retain the three-dimensional architecture, and are suitable to investigate sexually dimorphic (patho)physiological processes (21). Since both the human and rat placenta are hemochorial, they share important structural similarities, making the rat placenta a good model for studying placental physiology. Therefore, in this study, we hypothesized that *ex vivo* cultured precision-cut slices of the rat placenta represent an alternative experimental approach for studying sex-dependent inflammatory responses and inflammation-associated pathologies in the placenta (**Figure 1**). We used both bacterial (lipopolysaccharide/LPS) and viral (Polyinosinic:polycytidylic acid/Poly I:C) agents to induce inflammation and analyzed i) tissue viability and integrity, ii) the expression levels of proinflammatory genes and proteins in the male and female placentas, and iii) the effect of anti-inflammatory agents on the placental inflammatory response.

## Materials and methods

### Reagents

Lipopolysaccharides from Escherichia coli O111:B4 (LPS; product #: L2630), Polyinosinic–polycytidylic acid sodium salt (Poly I:C; product #: P1530), thiazolyl blue tetrazolium bromide (MTT; product #: M5655), gentamycin (product #: G1397), curcumin (product #: C7727), 1α,25-dihydroxyvitamin D3 (product #: D1530), and progesterone (product #: PHR1142) were purchased from Sigma-Aldrich (St. Louis, MO, USA). Tri Reagent solution was obtained from the Molecular Research Centre (Cincinnati, OH, USA). All other chemicals were of analytical grade.

### Animals

Pregnant Wistar rats (10-14 weeks old) were purchased from Velaz, Ltd (Czech Republic). The animals were kept in cages, with constant room temperature, low noise, and standard conditions of 12L:12D. All rats received food and water *ad libitum*. Gestation day 1 (Gd1) was established by the presence of a copulatory plug of sperm after overnight mating. On Gd 20, rats were anesthetized with pentobarbital in a dose of 40 mg/kg administered into the tail vein. The gestational sacs were separated, and 4 male and 4 female placentas (determined optically by measuring the anogenital distance in the fetuses) were separately collected from each dam in Krebs-Henseleit buffer [prepared as described previously (22)]. Subsequently, fetal sex was confirmed by end-point PCR analysis (see below). The n number represents the number of dams.

Experiments were approved by the Ethical Committee of the Faculty of Pharmacy in Hradec Kralove (Charles University, Czech Republic); they were carried out in accordance with the Guide for the care and use of laboratory animals (1996) and the European Convention for the protection of vertebrate animals used for experimental and other scientific purposes.

### Preparation and culture of rat placenta slices ex vivo

Placental slicing (**Figure 1**) was performed using a protocol by Gilligan et al. (21). The whole placenta was embedded with 3% low-gelling agarose, and the temperature of agarose was maintained between 36.5°C and 37°C to avoid destruction of placental tissues. Once the agarose solidified, the agarose-embedded placenta was transferred to Krumdiec tissue slicer (Alabama R&D, Munford, AL, USA), and placental slices were generated with a thickness of 200 µm (moderate speed; 4 slices/minute). Approximately 40-50 mg of intact slices were cultured in 24-well plates (TPP, Switzerland) containing 1 ml of Dulbecco’s Modified Eagle Medium (DMEM) supplemented with 10% fetal bovine serum (FBS), 100 U/ml penicillin, 0.1 mg/ml streptomycin, and 50 µg/ml gentamycin. The slices were maintained under standard cell culture conditions, 21% O_2_, 5% CO_2_ at 37°C in a sterile incubator, and fresh medium was replaced every 24 hours.

### Treatment of placenta slices with LPS, Poly I:C, and anti-inflammatory agents

Placental slices were cultured for 36 hours under normal conditions (stabilization period) and then exposed to various concentrations of LPS (0.1, 1, 5 µg/ml) or Poly I:C (1, 10, 50 µg/ml) for 4 and 18 hours. In a separate set of experiments, the anti-inflammatory agents, curcumin, 1α,25-dihydroxyvitamin D3 or progesterone, were dissolved in DMSO (at final DMSO concentrations of 0.04, 0.2, and 0.03%, respectively). Prior to LPS treatment, placental slices were preincubated individually with curcumin (25 µM), 1α,25-dihydroxyvitamin D3 (100 nM) and progesterone (1 µM) for 4 hours. Subsequently, the slices were exposed to a 4-hour treatment with LPS (5 µg/ml) and the respective anti-inflammatory agents. Experiments with anti-inflammatory agents were conducted in absence of FBS and antibiotics. After the treatment, the tissue was snap-frozen, and the culture media was collected (**Figure 1**). Samples were stored at -80°C until further analysis.

### Lactate dehydrogenase activity

The integrity of the slices was tested by measuring LDH release into the culture media using a colorimetric LDH activity assay kit (product #: MAK066; Sigma-Aldrich, St. Louis, MO, USA) according to the manufacturer’s instructions. Absorbance was measured at 450 nm in a 96-well plate using the Hidex Sense β Plus microplate reader (Hidex, Finland). LDH activity in the media was normalized to the tissue weight.

### MTT assay

Viability of the slices was assessed by measuring the MTT (3-(4,5-dimethylthiazol-2-yl)-2,5-diphenyltetrazolium bromide) reduction assay. To evaluate the incorporation of MTT into the tissue, placental slices were incubated with 1 mg/ml of MTT (in phenol red-free medium) at 37° C for 2 hours. Subsequently, the slices were placed in DMSO and incubated for 5 minutes to release the formazan crystals. The absorbance was measured at 570 nm and 690 nm using the Hidex Sense β Plus microplate reader (Hidex, Finland). Results are expressed as the difference between 570 and 690 nm absorbance, normalized to the tissue weight.

### RNA extraction and quantitative polymerase chain reaction analysis

Total RNA was extracted from placental slices using TRI reagent (Molecular Research Center, Cincinatti, OH, USA) according to the manufacturer’s instructions. RNA concentration and purity were determined using a NanoDrop™ 1000 spectrophotometer (Thermo Fisher Scientific, Waltham, MA, USA). cDNA was synthesized using the iScript Advanced cDNA synthesis kit (Bio-Rad, Hercules, CA, USA) according to the kit protocol. qPCR analysis for inflammatory genes was carried out in triplicates using the Taqman™ Universal Master Mix II, no UNG (Thermo Fisher Scientific, Waltham, MA, USA). The samples were amplified in a 5 µl reaction volume (384-well plate format) and following the thermal conditions recommended by the manufacturer. The expression of the target genes was calculated by the ΔΔCT method (23), and the relative expression was normalized to the geometric mean of beta-2 microglobulin (*B2m*), TATA-binding protein (*Tbp*), and tyrosine 3-monooxygenase/tryptophan 5-monooxygenase activation protein zeta (*Ywhaz*). The following primers were purchased from Thermo Fisher Scientific (Waltham, MA, USA) and used in our study: *Tnf-α* (Rn99999017_m1), *Il-6* (Rn01410330_m1), *Il-1β* (Rn00580432_m1), *Ifn-γ* (Rn00594078_m1), *Il-10* (Rn00563409_m1), *Tlr3* (Rn01488472_g1), *Tlr4* (Rn00569848_m1), *Ywhaz* (Rn00755072_m1), *B2m* (Rn00560865_m1) and *Tbp* (Rn01455646_m1).

### Fetal sex confirmation by end-point PCR analysis

End-point PCR analysis was used to confirm fetal sex as previously described (24). Briefly, genomic DNA was isolated using TRI reagent, and PCR amplification was performed using the Bio-Rad T100™ Thermal Cycler (Hercules, CA, USA). X-chromosome-specific and Y-chromosome-specific amplicons were analyzed on a 1.5% agarose gel using the HyperLadder™ 100 bp length marker (Bioline, Taunton, MA, USA). Ultraviolet light imaging was performed using the ChemiDoc MP detection system (Bio-Rad, Hercules, CA, USA).

### Cytokine quantification by ELISA

The concentrations of proinflammatory cytokines TNF-α (catalogue #: ERA56RB), IL-6 (catalogue #: ERA31RB), and IL-1β (catalogue #: BMS630) were measured in the cell-free culture media using commercially available ELISA kits from Thermo Fisher Scientific (Waltham, MA, USA), according to the manufacturer’s instructions. The cytokine concentration in the media was normalized to the tissue weight.

### Statistical analysis

Statistical analyses were implemented in GraphPad Prism 8.3.1 software (GraphPad Software, Inc., San Diego, USA) using two-way ANOVA followed by Dunnett’s multiple comparison tests. All experiments were performed in 3-5 biological and 3 technical replicates. The n number represents the number of dams from which 4 male and 4 female placentas were collected to generate the slices. Data are presented as mean ± SEM and results were considered significant when p < 0.05.

## Results

### Viability and integrity of placental slices during cultivation and treatment

**Figure 2:**
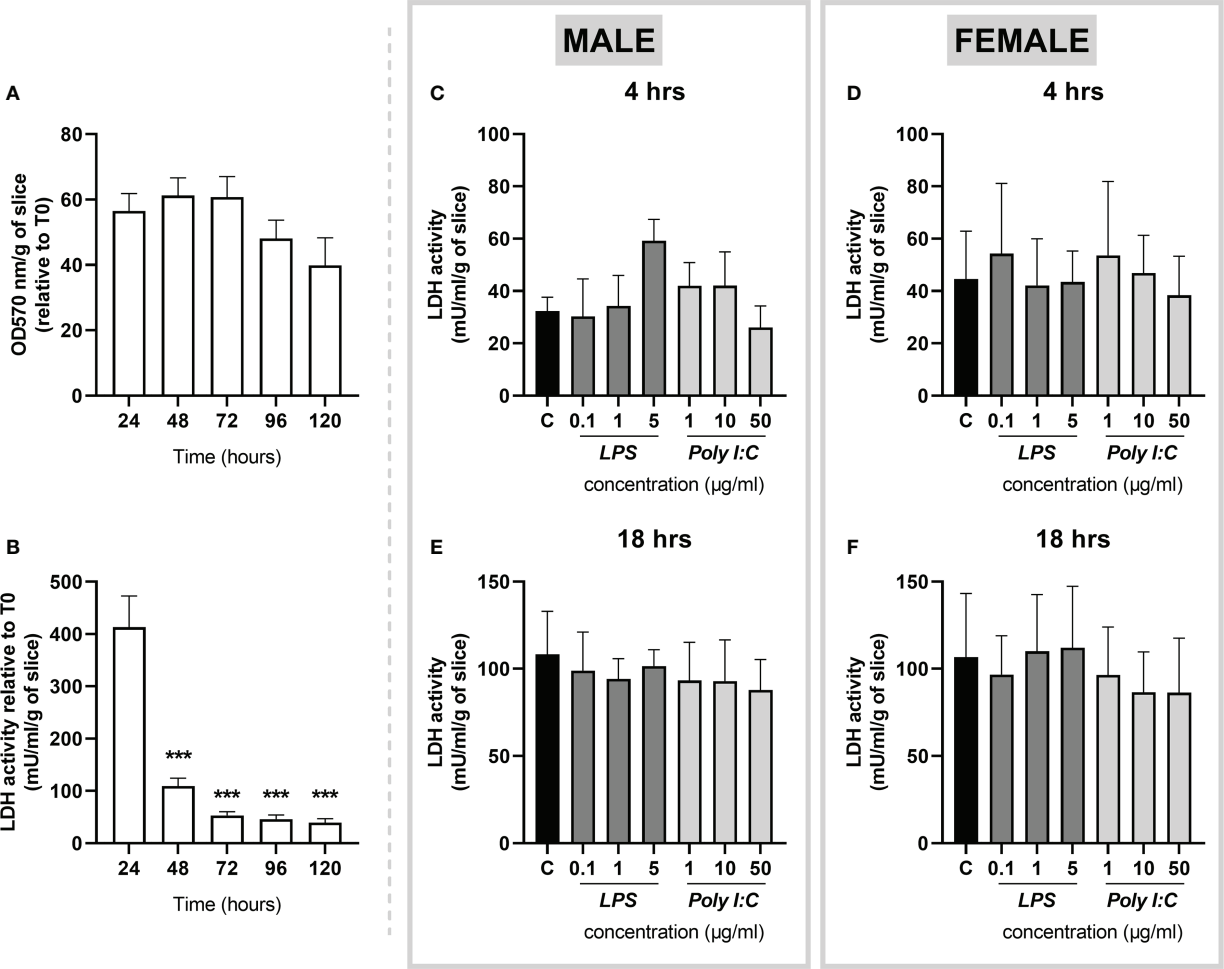
Viability and integrity of precision cut slices prepared from rat term placenta. **(A)** Viability of placenta slices was assessed by the MTT assay every 24 hours over a 120-hour period. **(B)** To evaluate tissue integrity, placenta slices were cultured for 120 hours under basal conditions, and LDH release was measured in the culture media every 24 hours. Similarly, LDH activity was evaluated upon LPS and Poly I:C treatment in male **(C, E)** and female **(D, F)** placental slices for 4 and 18 hours. All results were normalized to the tissue weight. Data represent the mean ± SEM; n ≥ 3. ***P < 0.001.

The viability of the rat placental slices was determined by the MTT assay, indicating no significant reduction in slice viability for up to 120 hours (**Figure 2A**). In addition, the integrity of placental slices was determined by the release of LDH into the culture media. At 24 hours, LDH activity was approximately 400 mU/ml/g and gradually decreased to the baseline levels (< 50 mU/ml/g) and remained stable for up to 120 hours (**Figure 2B**). Subsequently, to determine whether LPS and Poly I:C treatment exert cytotoxic effects on the placental slices, LDH activity was determined in treated samples at 4 and 18 hours. We observed no significant increase in LDH release in the culture media of LPS/Poly I:C-exposed slices (**Figures 2C-F**).

Taken together, the above-described findings show that rat placental slices are viable for up to 120 hours of culturing under basal conditions. Importantly, the slices remain viable upon treatment with LPS and Poly I:C at indicated concentrations and treatment times.

### Effect of LPS and Poly I:C on proinflammatory cytokine response in placental slices

**Figure 3:**
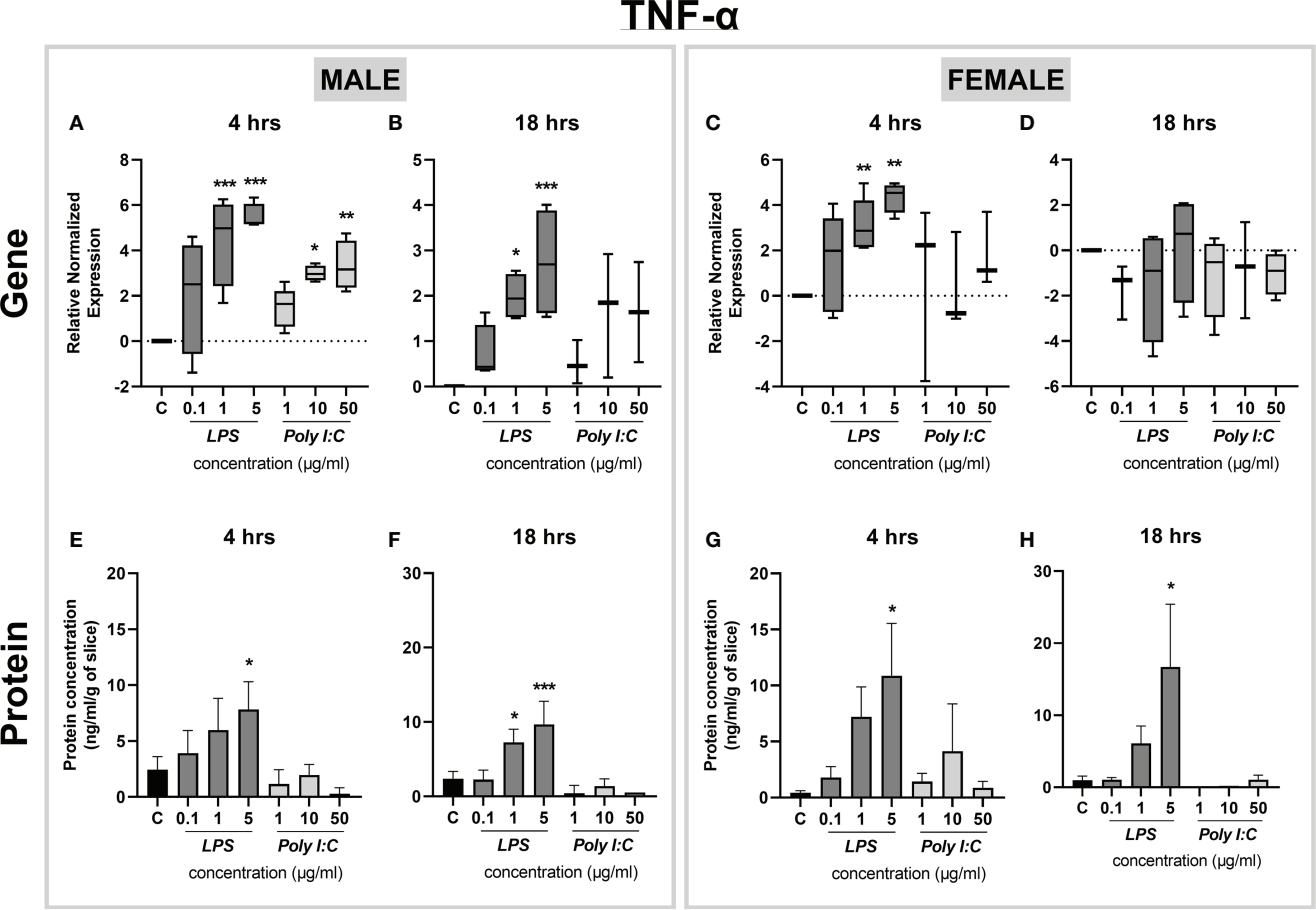
Effect of LPS and Poly I:C on TNF-α gene expression and protein concentration. Male and female placental slices were exposed to LPS and Poly I:C at various concentrations for 4 and 18 hours. Gene expression of *Tnf-α* in placental slices was evaluated by qPCR analysis **(A-D)**. TNF-α protein concentrations were measured in culture media using a commercially available ELISA kit **(E-H)**. Data are shown as Tukey boxplots or mean ± SEM; n ≥ 3. *P < 0.05; **P < 0.01; ***P < 0.001.

Exposure of rat placental slices to different concentrations of LPS (0.1, 1, and 5 µg/ml) or Poly I:C (1, 10, and 50 µg/ml) for 4 and 18 hours triggered alterations in the gene expression and protein secretion of several proinflammatory cytokines. We found that gene expression of *Tnf-α* was significantly increased in both male (**Figure 3A**) and female (**Figure 3C**) placentas treated with LPS (1 and 5 µg/ml) for 4 hours. This effect was observed in the male placentas also at 18 hours of treatment (**Figure 3B**), while in the female placenta, *Tnf-α* expression returned to baseline expression levels (**Figure 3D**). On the other hand, Poly I:C treatment caused *Tnf-α* upregulation only in male placental slices treated for 4 hours (**Figure 3A**).

To confirm changes in gene expression also at the protein level, we applied ELISA analysis to quantify TNF-α released in the culture media. We found that treatment with 5 µg/ml of LPS significantly induced TNF-α protein release in the culture media, and this effect was evident for both sexes and treatment times (**Figures 3E-H**). Interestingly, the tissue response at 18 hours of treatment with 5 µg/ml of LPS showed sex-dependent changes in the degree of TNF-α protein upregulation. While in the male placenta the protein concentration was increased by an average of 4-fold (**Figure 3F**), in the female placenta we observed an average of 17-fold upregulation of TNF-α concentration, compared to basal levels (**Figure 3H**).

**Figure 4:**
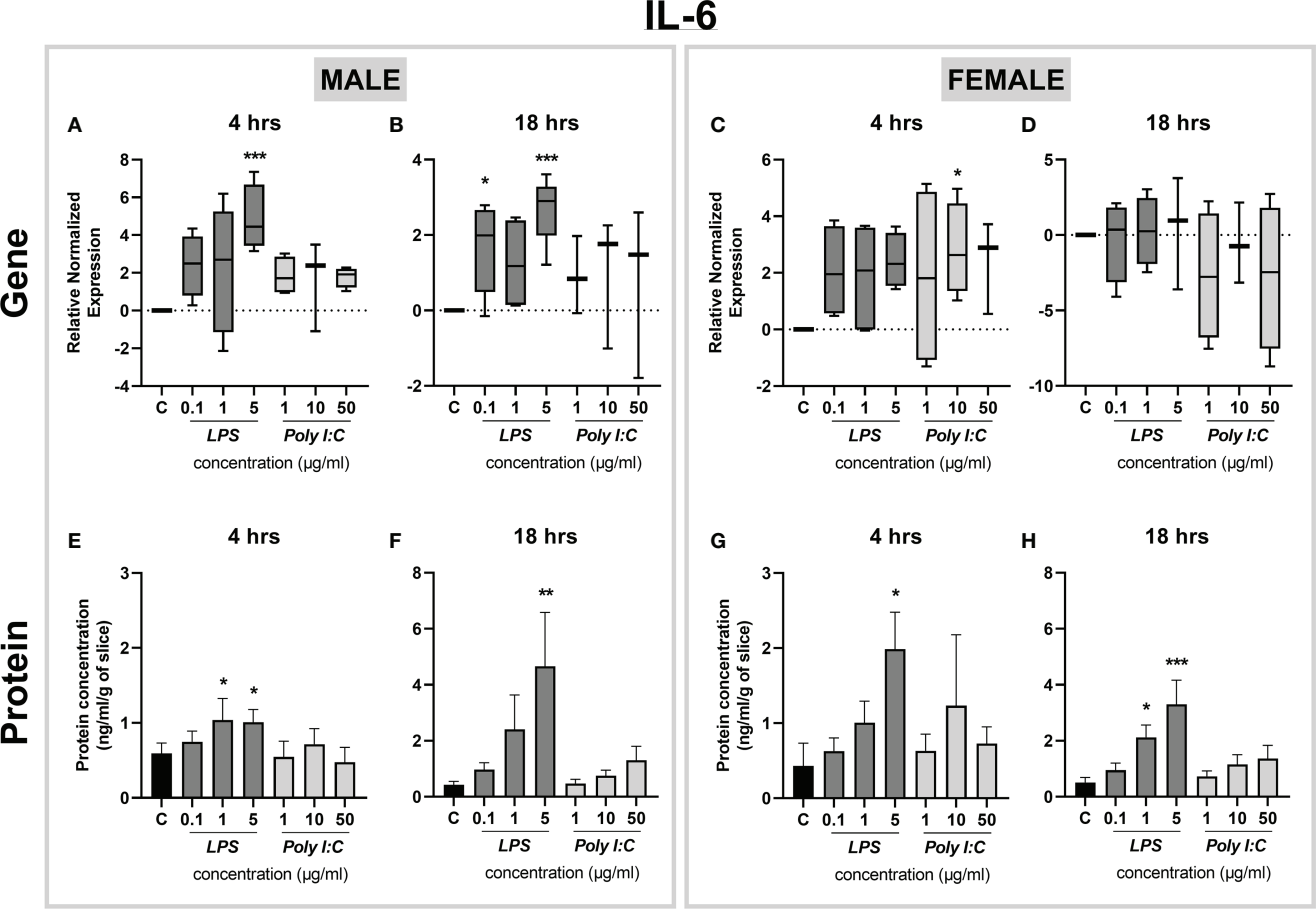
Effect of LPS and Poly I:C on IL-6 gene expression and protein concentration. Male and female placental slices were exposed to LPS and Poly I:C at various concentrations for 4 and 18 hours. Gene expression of *Il-6* in placental slices was determined by qPCR analysis **(A-D)**. IL-6 protein concentrations were measured in culture media using a commercially available ELISA kit **(E-H)**. Data are shown as Tukey boxplots or mean ± SEM; n ≥ 3. *P < 0.05; **P < 0.01; ***P < 0.001.

Similarly, we observed LPS-induced transcriptional alterations in the expression of *Il-6*. Specifically, *Il-6* gene expression was significantly higher in male placentas exposed to 5 µg/ml LPS at both time points tested (**Figures 4A, B**), while placental slices prepared from female placentas were less responsive to the treatment (**Figures 4C, D**). On the other hand, IL-6 protein release was significantly higher in both male and female placental slices treated with 5 µg/ml LPS for 4 and 18 hours (**Figures 4E-H**). No changes in IL-6 gene expression or protein concentrations were observed after the treatment of placenta slices with Poly I:C (**Figures 4A-H**).

**Figure 5:**
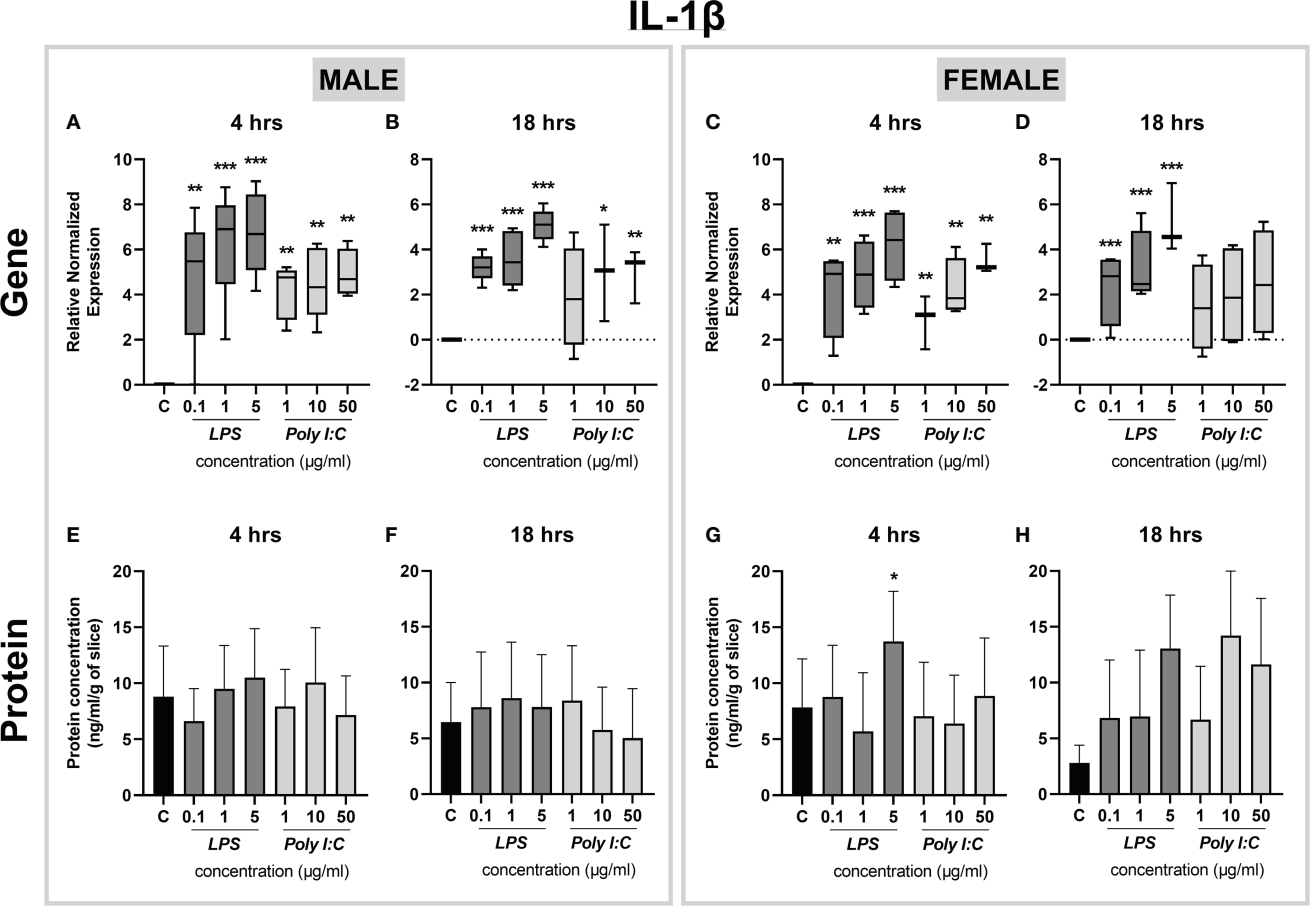
Effect of LPS and Poly I:C on IL-1β gene expression and protein concentration. Male and female placental slices were exposed to LPS and Poly I:C at various concentrations for 4 and 18 hours. Gene expression of *Il-1β* in placental slices was evaluated by qPCR analysis **(A-D)**. IL-1β protein concentrations were measured in culture media using a commercially available ELISA kit **(E-H)**. Data are shown as Tukey boxplots or mean ± SEM; n ≥ 3. *P < 0.05; **P < 0.01; ***P < 0.001.

Next, the expression of IL-1β was determined, revealing changes only at the level of gene expression. *Il-1β* mRNA was significantly increased in both male and female placental slices treated with increasing concentrations of LPS for 4 and 18 hours (**Figures 5A-D**). Moreover, Poly I:C exerted transcriptional upregulation of *Il-1β* in both male and female placental slices (**Figures 5A-C**). While in male placentas, this effect was observed even at 18 hours of Poly I:C exposure (**Figures 5A, B**), female placentas were responsive only at 4 hours of treatment (**Figure 5C**). On the other hand, the protein concentration of IL-1β was not affected by either treatment (**Figures 5E-H**), except for the female placenta exposed to a higher concentration of LPS (5 µg/ml) for 4 hours (**Figure 5G**). It is also worth noting that at basal levels (when no treatment was applied), rat placental slices release different contents of cytokines in the following order: IL-1β > TNF-α > IL-6 (**Figures 3**–**5**). Thus, longer treatment may be necessary to observe distinguishable effects on IL-1β protein secretion. In addition, we examined the expression of *Ifn-γ* and the anti-inflammatory cytokine *Il-10* at the mRNA level. We observed no significant changes in *Ifn-γ* gene expression upon treatment (**Supplementary Figure 1A-D**). On the contrary, *Il-10* expression was significantly upregulated in LPS (1 and 5 µg/ml) treated male and female placental explants at both 4 and 18 hours (**Supplementary Figure 1E-H**).

Altogether, our results show that the exposure of placental slices to different concentrations of LPS leads to a significant increase in the expression of proinflammatory cytokines at both the gene and protein levels. On the other hand, rat placental slices are generally less responsive to the Poly I:C treatment used in this study.

### Gene expression of *Tlr3* and *Tlr4* in placental slices stimulated by Poly I:C and LPS

**Figure 6:**
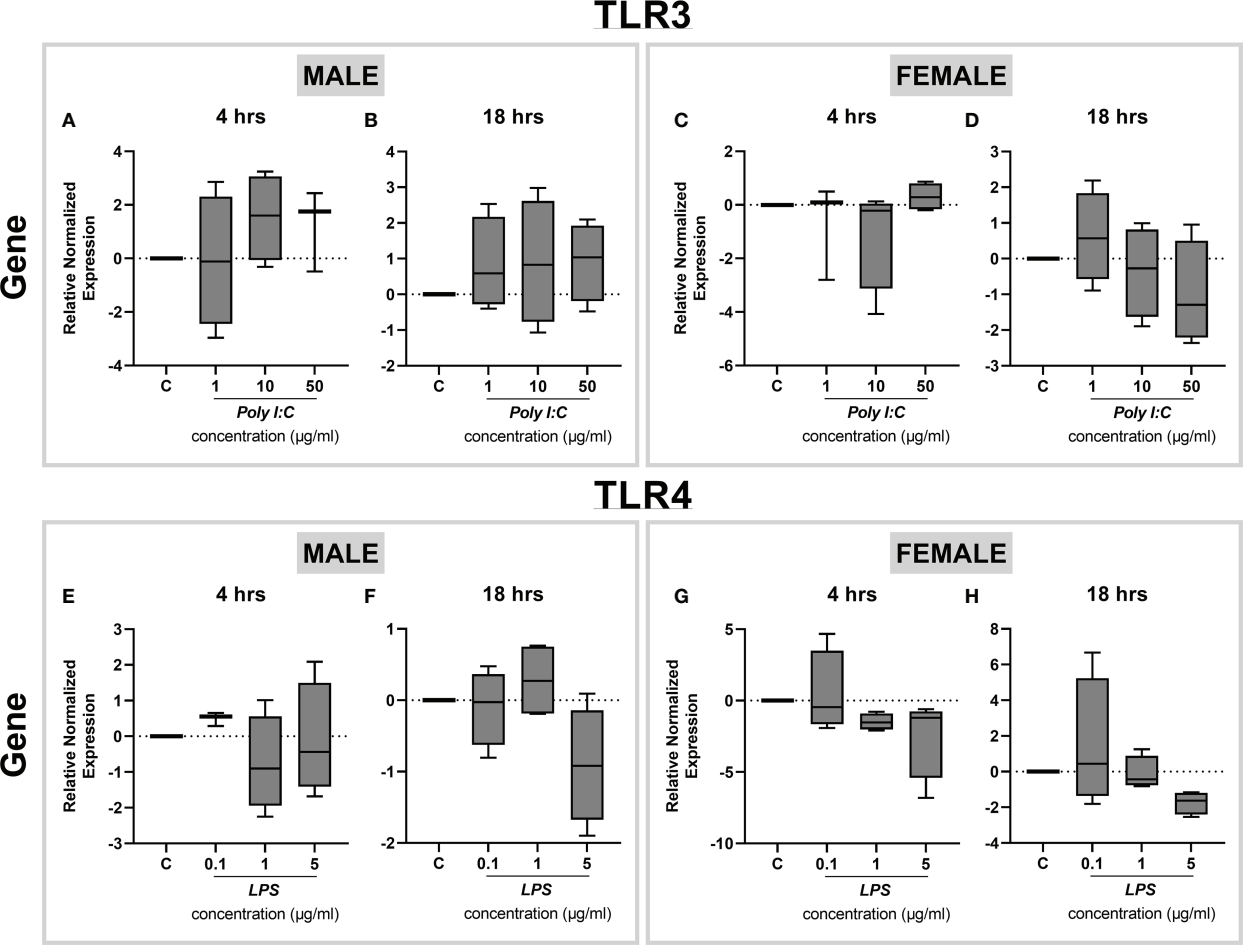
Effect of LPS treatment on *Tlr3* and *Tlr4* expression in rat placental slices. Male and female placentas were exposed to increasing concentrations of Poly I:C **(A-D)** and LPS **(E-H)** for 4 and 18 hours, and the gene expression of *Tlr3* **(A-D)** and *Tlr4* **(E-H)** was determined by qPCR analysis. Data are shown as Tukey boxplots, n=4.

Since toll-like receptor 3 (TLR3) and 4 (TLR4) recognize Poly I:C and LPS as ligands, respectively, we next examined the expression profiles of *Tlr3* and *Tlr4* genes in rat placental slices exposed to various concentrations of Poly I:C and LPS. We found that *Tlr3* gene expression is not altered upon Poly I:C treatment for 4 and 18 hours (**Figures 6A-D**). Similarly, stimulation of rat placental slices by LPS did not exert any regulatory effects on *Tlr4* gene expression (**Figures 6E-H**). This was true for both male and female placentas.

### Effect of anti-inflammatory compounds on LPS-induced inflammation in rat placental slices

**Figure 7:**
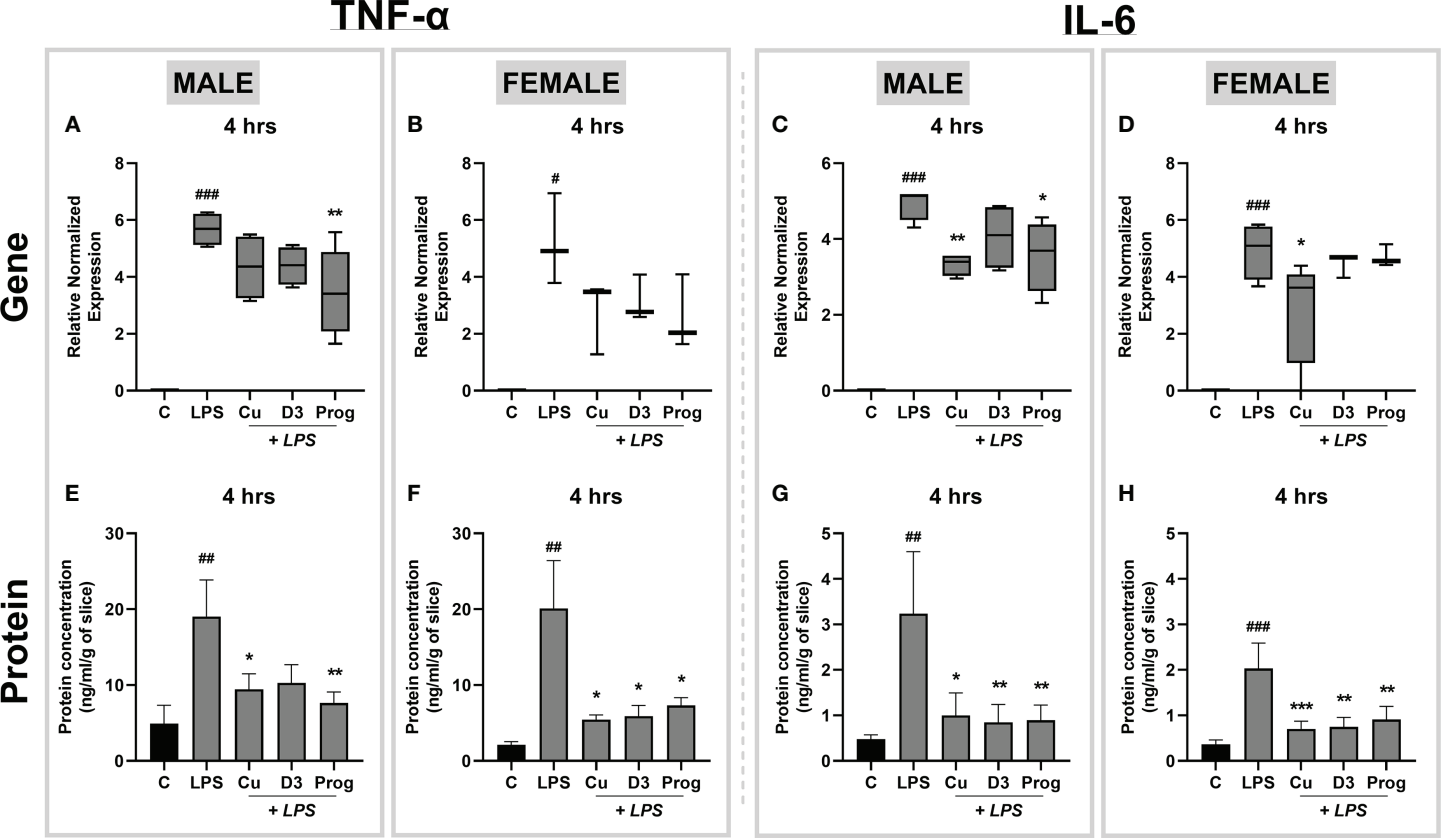
Effect of curcumin, 1α,25-dihydroxyvitamin D3, and progesterone on LPS-induced inflammation in the rat placental slices. Following a 4-hour preincubation phase with inhibitors, male and female placental slices were treated with LPS (5 µg/ml) in the presence and absence of curcumin, 1α,25-dihydroxyvitamin D3, or progesterone for an additional 4 hours. Gene expression of proinflammatory cytokines was subsequently determined by qPCR analysis **(A-D)**, while cytokine release in the culture media was analyzed by ELISA **(E-H)**. Data are shown as Tukey boxplots or mean ± SEM; n ≥ 4. *P < 0.05; **P < 0.01; ***P < 0.001; ^#^P < 0.05; ^##^P < 0. 01; ^###^P < 0. 001. # compared to the control; *compared to LPS.

To characterize LPS-induced inflammation *via* TLR4-mediated signaling pathways in rat placental slices, we next evaluated the anti-inflammatory effect of curcumin, 1α,25-dihydroxyvitamin D3 (the active form of vitamin D3) and progesterone. All these compounds are reported as TLR4 inhibitors and anti-inflammatory agents; the concentrations used in this study were based on previously published studies (25–29). Placental slices were initially preincubated with antiinflammatory compounds of interest for 4 hours, and then exposed to 5 µg/ml LPS in the presence or absence of inhibitors for 4 hours. The anti-inflammatory effect was examined by evaluating the TNF-α and IL-6 gene expression and protein release in the culture media. 1 µM progesterone significantly inhibited LPS-induced *Tnf-α* and *Il-6* gene expression in the male placenta (**Figures 7A, C**), while this effect was not observed in the female placental slices (**Figures 7B, D**). On the other hand, 25 µM curcumin significantly reduced *Il-6* (but not *Tnf-α*) gene expression in both male and female placental slices (**Figures 7C, D**).

In contrast, ELISA analysis of cytokine release in culture media demonstrated that all three anti-inflammatory agents significantly inhibited LPS-induced TNF-α and IL-6 concentrations in both male and female placentas (**Figures 7E-H**). To confirm that this effect targets the LPS-induced cytokine release, separate experiments were carried out only in the presence of the anti-inflammatory agents (and in the absence of LPS). Our results demonstrate that anti-inflammatory drug treatment alone had no effect on TNF-α and IL-6 gene and protein expression (**Supplementary Figure 2**). Together, these findings indicate that the precision-cut slices are a suitable model for assessing the effects of anti-inflammatory drugs on LPS-induced inflammation in the placenta.

## Discussion

Precision-cut slices are viable tissue explants that have been used to investigate metabolism, transport, and therapeutic targeting in the liver, lung, and intestinal tract (16–18, 30). However, relatively limited research has been conducted on placental slices since their introduction by Gilligan et al. in 2012 (21). This is the first report to use the precision-cut slice model to investigate acute inflammatory response in the rat placenta. We show that proinflammatory cytokines are activated in placental slices exposed to the TLR4 ligand, LPS, in a sex-dependent manner, and this effect can be mitigated by TLR4 inhibitors.

In tissue culture models, cell viability is of primary importance. Consistent with Gilligan et al. (21), we show that at basal culturing conditions, LDH release (used as a marker of tissue injury) reaches the highest levels in the first 24 hours of culture. Nonetheless, LDH release was significantly reduced after 48 hours, and its concentrations were maintained at the base level for up to 120 hours. Correspondingly, cellular metabolic activity analyzed by the MTT assay revealed no significant reduction in placental slice viability for up to 120 hours. In line with previous observations (18, 21, 31), this indicates compromised tissue integrity during the first 24 hours after the slicing procedure and necessitates the use of a recuperation period before the experiments are initiated. Therefore, in this study, all treatment studies were conducted 36 hours after slice preparation. Subsequently, to ensure that LPS and Poly I:C do not exert cytotoxic effects in the rat placental slices, we evaluated LDH release after treatment for 4 and 18 hours. We demonstrate that LPS and Poly I:C do not result in tissue damage in placental slices at the concentrations and exposure times utilized in our study. Therefore, any tissue response observed in the following experiments is solely a result of the proinflammatory processes induced by LPS and Poly I:C.

It is increasingly evident that inflammation during pregnancy stimulates a hostile proinflammatory environment in the fetoplacental unit, characterized by a surge in the release of cytokines (7, 32). A preclinical placental inflammatory model is a powerful tool to identify the tissue response to immune-activating agents and analyze the downstream effects of the proinflammatory cytokine network. Previous studies in other placental models have reported elevated levels of proinflammatory cytokines (particularly TNF-α, IL-6, and IL-1β) after treatment with LPS and Poly I:C (33–36). However, to date, the use of precision-cut slices in studying inflammatory responses has only been tested in the liver and lung slices from piglets and mice (19, 20). Here we show that exposure of rat placental slices to LPS leads to a significant increase in the expression and secretion of several key proinflammatory cytokines, TNF-α, IL-6, and IL-1β. This suggests the activation of the TLR4-dependent NF-κB pathway, which results in the induction of inflammatory genes (34, 37). At the same time, we observed a significant increase in gene expression of the anti-inflammatory *Il-10*, indicating the activation of TRIF-dependent MAPK and STAT pathways (38, 39). To further investigate LPS actions on TLR4 in rat placental slices, we examined the expression of *Tlr4* gene upon treatment with increasing concentrations of LPS. Previous studies have shown contradicting data; while some showed no effect of LPS on TLR4 expression (33–35) others reported upregulation of placental TLR4 upon LPS stimulation (37, 40). Here we observed that *Tlr4* gene expression remains unaltered regardless of the LPS concentration, time of exposure or fetal sex. Thus, the resulting induction of cytokines in placental slices is likely due to the activation of the TLR4 downstream signaling pathway.

On the other hand, Poly I:C stimulation resulted in a less pronounced inflammatory milieu, however, with considerable upregulation of proinflammatory cytokine gene expression in both male and female placentas. As Poly I:C targets TRL3, we investigated whether the Poly I:C hypo-responsiveness observed in our studies could be due to the regulation of *Tlr3* gene expression in rat placental slices treated with Poly I:C. Nonetheless, we detected no changes in *Tlr3* gene expression regardless of the dose, exposure time or fetal sex. Since the placenta demonstrates a greater response to infection in earlier stages of pregnancy compared to term (41), we speculate that the gestational age chosen for our studies (term placenta) may play a role in the magnitude of immune response. In addition, endotoxin contamination and molecular weight differences in Poly I:C composition were reported to cause considerable variability in immune response *in vitro* and *in vivo* in rats (42, 43). These factors could have affected our results in Poly I:C experiments and will be subjected to further studies.

To validate the suitability of precision-cut rat placental slices for anti-inflammatory and mechanistic studies, we next evaluated the efficacy of several TLR4 inhibitors, curcumin, 1α,25-dihydroxyvitamin D3 and progesterone (25–29) to ameliorate LPS-induced inflammation. Our results showed that all tested anti-inflammatory compounds significantly lowered the release of proinflammatory cytokines TNF-α and IL-6 into the culture media. These findings further support the use of rat placental slices as an apt model to investigate TLR4-induced inflammatory pathways and identify possible targets for therapy.

One of the main advantages of the rat placental slice model is the ability to address sex-dependent processes by generating slices from both male and female placentas obtained from the same dam. So far, conflicting reports have been published regarding sexual dimorphism in the placental inflammatory response. Recent studies have indicated that the male placenta responds to LPS in a more profound manner, with higher levels of the proinflammatory cytokines TNF-α and IL-6 (12, 13, 44, 45). Others, however, have demonstrated that the female placenta shows a stronger proinflammatory response to various inflammatory stimuli (14, 15, 46). In our study, we observed that placental slices prepared from the female placentas displayed a higher sensitivity to LPS resulting in a larger secretion of proinflammatory cytokines, particularly TNF-α. This could be due to the level of sex hormones (estrogens and testosterones) produced by the placenta; while estrogen acts as an immune enhancer by inducing NF-κB signaling, testosterone is generally considered an immune suppressor (13, 14). In addition, several studies have shown a higher expression of cytokine receptors in female placentas than in male placentas (13–15). However, future studies are required to decipher the molecular mechanisms behind placental sexual dimorphism.

Innate immune responses are orchestrated by a diverse range of maternal and fetal cells at the decidual-placental interface (47). Hence, rat placental slices represent an advantageous model for immune studies since they maintain the multicellular architecture of the tissue, cell-cell interactions, and the three-dimensional structure. At the same time, the slicing procedure does not require the use of proteolytic enzymes, thus reducing tissue damage. Moreover, placentas from diseased pregnancies can be used to study pathological processes or investigate the therapeutic effects of drugs. Lastly, preparing placental slices from the rat placenta complies with two main 3R principles (17, 48) since the high-throughput nature of the model can significantly reduce the number of animals used for experiments. At the same time, refinement is achieved by minimal discomfort to the animals since all manipulations (culture, treatment) are performed ex vivo (17, 48). However, the model of rat placental slices also possesses certain limitations. Species differences must be considered, and the model’s applicability to specific biological processes must be carefully addressed to ensure translatability in humans. In addition, while the slices can be maintained in culture for up to 4 days, this is still a short time to evaluate chronic xenobiotics exposure.

In summary, we show that the precision-cut slices prepared from the rat placenta represent a valuable tool to i) study inflammatory processes induced by bacterial (LPS) or viral (Poly I:C) agents, ii) target TLR4 activation, and iii) investigate the effects of sexual dimorphism on immune activation. Since maternal inflammation is an important factor associated with poor pregnancy outcomes and fetal morbidity, this model can help elucidate placental pathways involved in fetal programming and has potential application in the field of immunology, pharmacology, and toxicology.

## Data availability statement

The original contributions presented in the study are included in the article/**Supplementary Material**. Further inquiries can be directed to the corresponding author.

## Ethics statement

The animal study was reviewed and approved by Ethical Committee of the Faculty of Pharmacy in Hradec Kralove, Charles University, Czech Republic.

## Author contributions

Study concept and design - KA, IV, FS. Data acquisition - KA, CA, TS, MV-N, BB, IV. Data analysis - KA, RK. Data interpretation - KA, CA, RK, FS. Manuscript preparation - KA, CA, RK, FS. All authors have approved the submitted version and are personally accountable for their contributions to the manuscript.
