# Supplementary material for: Precision-cut rat placental slices as a model to study sex-dependent inflammatory response to LPS and Poly I:C
Source: Front Immunol. 2022 Dec 20;13:1083248. doi: 10.3389/fimmu.2022.1083248 (PMC9807759; doi:10.3389/fimmu.2022.1083248)
Supplement: Supplementary file 1 [file DataSheet_1.docx]

Supplementary Material

**
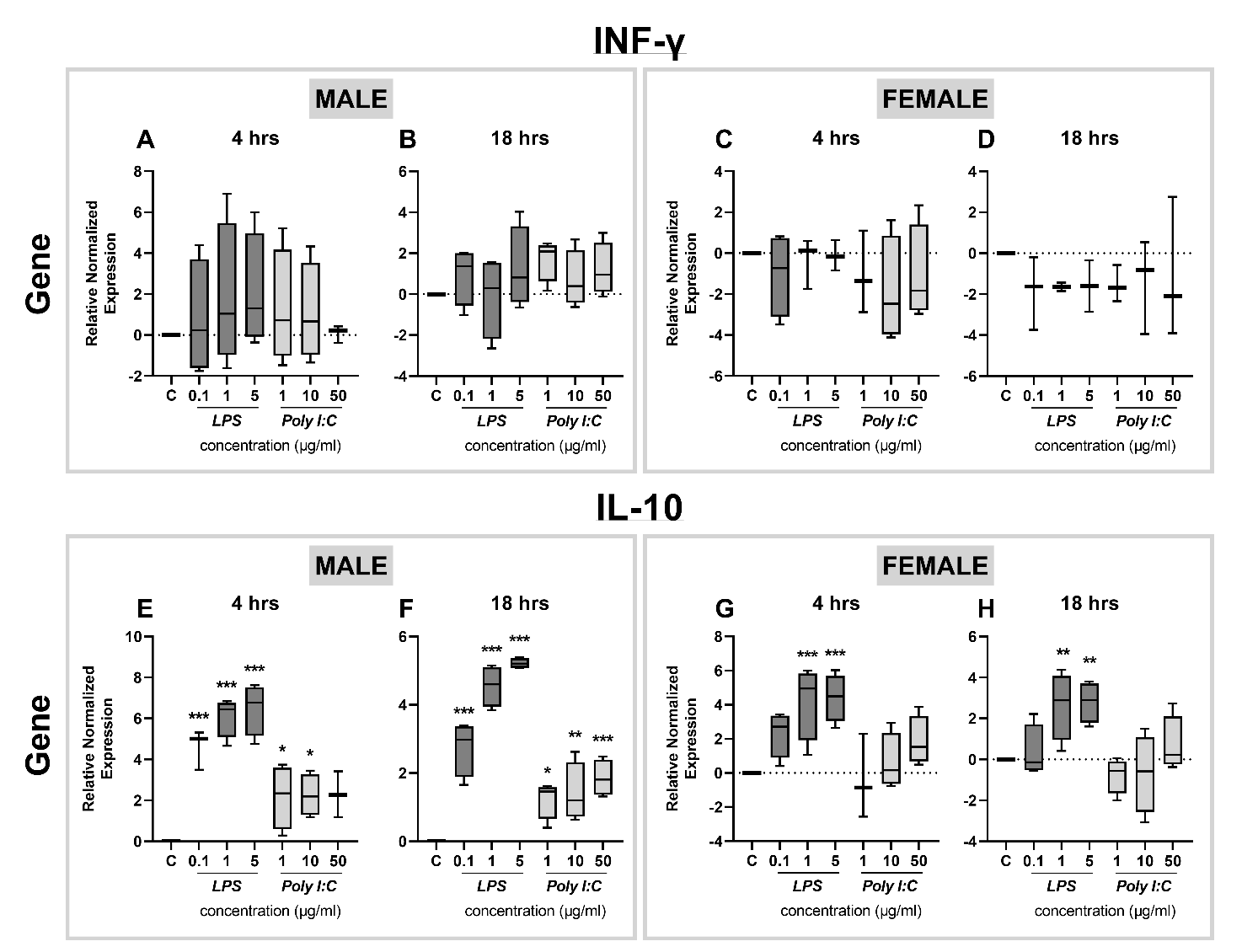
**

**Figure S1:** Effect of LPS and Poly I:C on IFN-γ and IL-10 gene expression. Male and female placental slices were exposed to LPS and Poly I:C at various concentrations for 4 and 18 hours and determined the level of gene expression of *Inf-γ* **(A-D)** and *Il-10* **(E-H)** by qPCR. Data are shown as Tukey boxplots, n ≥ 3. *P < 0.05; **P < 0.01; ***P < 0.001.


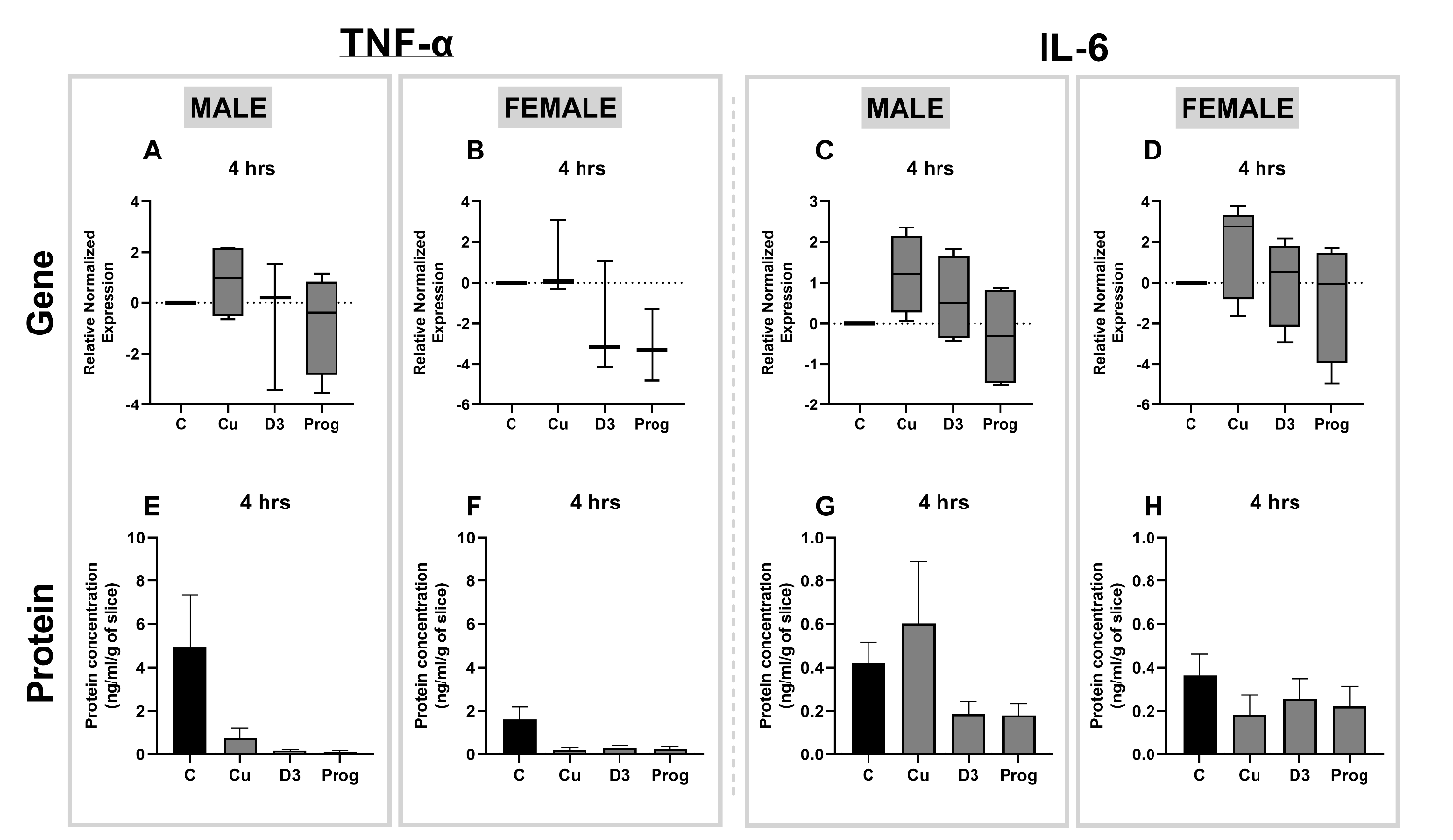


**Figure S2:** Effect of curcumin, 1α,25-dihydroxyvitamin D3 and progesterone on cytokine gene expression and protein concentration in rat placental slices. Male and female placental slices were treated with curcumin, 1α,25-dihydroxyvitamin D3 or progesterone for 4 hours. Gene expression level of *Tnf-α* and *Il-6* was determined by qPCR analysis (A-D), whereas their concentration in the culture medium was analyzed by ELISA (E-H). Data are shown as Tukey boxplots or mean ± SE; n ≥ 4.
